# Genetic mapping with testcrossing associations and F_2:3_ populations reveals the importance of heterosis in chilling tolerance at maize seedling stage

**DOI:** 10.1038/s41598-017-03585-0

**Published:** 2017-06-12

**Authors:** Jinbo Yan, Yu Wu, Wenming Li, Xiner Qin, Yi Wang, Bing Yue

**Affiliations:** 1 National Key Laboratory of Crop Genetic Improvement, Huazhong Agricultural University, Wuhan, China; 2 Industrial Crops Research Institution, Heilongjiang Academy of Land Reclamation of Sciences, Haerbin, China

## Abstract

Maize seedlings are sensitive to low temperatures, and genetic mapping for chilling tolerance at the seedling stage with genetically diverse populations would facilitate the genetic improvement of this important trait. In this study, quantitative trait loci (QTL) mapping for four chilling tolerance-related traits at the seedling stage was conducted via a genome-wide association study (GWAS) with 338 testcrosses. A total of 32 significant loci and 36 stress tolerance-related candidate genes were identified, though none of them have been revealed by QTL mapping using maize inbred lines in previous reports. Moreover, expression of ten of the candidate genes was induced by chilling stress in a maize hybrid, though only a few of these genes were upregulated in its tolerant parent. These implied that heterosis might be involved in maize chilling tolerance. To further evaluate the importance of heterosis in chilling tolerance at the seedling stage, genetic mapping for chilling tolerance was conducted using an F_2:3_ population derived from the two inbred lines used for the gene expression assay. Of the seven QTL revealed, six loci showed partial dominance or over-dominance effects. Results from this study demonstrate that heterosis plays an important role in chilling tolerance in maize seedlings.

## Introduction

Maize (*Zea mays* L.) is an important food, energy, forage and industrial crop. However, chilling (0–15 °C) has become a major environmental factor that limits maize production and its distribution. Chilling stress affects germination, seedling growth, early leaf development and overall maize crop growth and productivity^1^. In particular, maize seedlings are the most sensitive to chilling stress during the transition phase from heterotrophic to autotrophic growth^2^. Therefore, elucidating the mechanism of maize chilling tolerance at the seedling stage (3-leaf stage) will help to genetically improve this trait.

Yield performance is usually used to evaluate stress tolerance at the reproductive stage, while at the seedling stage, it mainly relies on morphological-physiological traits. Physiological traits, such as photosynthetic performance^3–5^, tissue water content, and levels of abscisic acid (ABA) and antioxidants^3^, usually respond to low temperatures earlier than changes in morphological traits and are extensively used as indicators of chilling tolerance. Additionally, as a kind of compatible solute to increase the osmotic potential and stabilize macromolecular structure, soluble sugar content can also be a good index for stress tolerance evaluation^6^. At the morphological level, chilling stress causes decreased growth rates, leaf elongation and dry weights^2^. Chilling stress decreases root hydraulic conductance^7^ and often results in water-stress symptoms. Root development in chilling-sensitive maize seedlings was found to be distinctly reduced under chilling stress, and the root-to-shoot ratio in chilling-sensitive inbred plants decreased under chilling stress conditions at the 2- to 3-leaf stage^8^. Because physiological traits are more difficult to investigate than morphological traits, the latter are usually used for germplasm evaluation in large-scale experiments.

With the development of molecular markers, a number of quantitative trait loci (QTL) for chilling tolerance have been identified by linkage mapping in maize^9–13^. Rapid linkage disequilibrium decay makes maize an excellent model crop for genome-wide association studies (GWAS), and a number of significant loci for some chilling tolerance-related traits have been recently detected by GWAS at the seedling stage in maize inbred lines^4, 5, 14^. In rice, the identification of genetic loci related to chilling tolerance at the seedling stage was also conducted using a GWAS^15^.

Hybrids are extensively used in maize and rice production, and they exhibit enhanced agronomic performance relative to their parents, especially for stress-related traits. Genetic factors contributing to the performance of some agronomic traits in hybrids are generally found to be different from their parents in crops^16, 17^, demonstrating that dissection of the genetic factors that contribute to heterosis is more important in maize and rice. Recently, Huang *et al*.^18^ identified a number of superior alleles that contribute to heterosis for 38 agronomic traits in rice via a GWAS with 1,495 rice hybrids. In maize, Revilla *et al*.^5^ also conducted a GWAS with testcrossing hybrids, though only one QTL for chilling tolerance was identified at the seedling stage. In this study, a number of significant loci and candidate genes for chilling tolerance were identified via GWAS at the seedling stage with a testcrossing association mapping panel; the QTLs were identified are completely different from those identified using maize inbred lines. Gene expression assays in the two inbred lines and their hybrids under chilling tolerance revealed that these candidate genes might be related to heterosis. Furthermore, genetic mapping with an F_2:3_ population derived from the inbred lines used for the gene expression assay confirmed the importance of heterosis in maize chilling tolerance at the seedling stage. The results from this study provide new insights into the principles of heterosis in chilling tolerance in maize seedlings.

## Results

### Trait performance

Four chilling tolerance-related traits, leaf rolling degree (LRD), water content in shoots and leaves (WCS), ratio of root-to-shoot (RRS), and soluble sugar content (SSC), were investigated. Performance of the four traits in the CMS (cytoplasmic male sterility) parent, S-Mo17, and the testcrosses is given in Table 1. Large variations were observed for all the traits. The performance of the traits in S-Mo17 was close to the trait means in the whole population. All the traits presented fit or closely fit to the normal distributions. This indicated that multiple genes are involved in the expression of these traits.

**Table 1:** Performance of leaf roll degree (LRD), water content of shoots and leaves (WCS), ratio of root-to-shoot (RRS), and soluble sugar content (SSC) in the testcrossing association mapping panel and the F_2:3_ population.

Traits	Parents^a^		Populations^b^				
	P_1_ ± SD	P_2_ ± SD	Mean	SD	Range	Skew	Kurt
**Testcrossing association mapping population**							
LRD	3.78 ± 0.18	—	3.54	1.11	1.00–5.00	−0.27	−0.87
WCS	87.88 ± 0.45	—	88.17	3.03	80.25–91.13	−0.60	1.20
RRS	1.03 ± 0.12	—	1.03	0.26	0.70–1.73	0.78	1.30
SSC	6.00 ± 0.33	—	5.29	2.11	2.84–10.61	0.69	0.19
**F**_**2:3**_ **population**							
LRD	2.75 ± 0.35	3.50 ± 0.71	3.31	0.82	1.00–5.00	0.16	0.34
WCS	—	85.71 ± 4.58	85.60	1.73	82.10–89.17	−0.12	−0.88
RRS	0.87 ± 0.04	1.80 ± 0.35	1.30	0.24	0.87–1.81	0.92	2.18
SSC	3.94 ± 0.43*	7.01 ± 0.41*	7.78	2.49	3.44–12.70	0.49	0.11

^a^P_1_ in the testcrossing association mapping population is S-Mo17; P_1_ and P_2_ in the F_2:3_ population are K932 and Mei C, respectively; “—”means the data is missing; SD = standard deviation; *represents the difference between the two parents is significant at the level of *p* < 0.05. ^b^Skew = skewness, Kurt = kurtosis.

In a germplasm evaluation test conducted in the field condition in the winter of 2014, Mei C and K932, which displayed different chilling tolerances at the seedling stage, were selected to construct an F_2:3_ population (Fig. S1). The variations in the F_2:3_ population as well as the phenotypic differences between their parents are also summarized in Table 1. Transgressive segregation was observed for all the traits in the population, except for RRS. The chilling tolerance in Mei C was better than K932 because it had higher values for LRD, RRS, and SSC. The difference between the two parents for SSC was significant at *p* < 0.05. All the traits presented also fit or closely fit to normal distributions.

Analysis of variance (ANOVA) of the data collected in the testcrossing association mapping panel and the F_2:3_ population indicated that variations due to genotype differences were significant for all the traits (0.00 < *p* < 0.03). In the testcrossing population, a significant positive correlation was detected between LRD and WCS (*r* = 0.37). However, the correlation coefficients among LRD, RRS, and SSC were negative (−0.50 < *r* < −0.01). The performance of the traits in different subpopulations was compared with the Student’s T-Test. In general, testcrosses derived from the temperate inbred lines performed better than that crossing with tropical and subtropical inbred lines for all the traits, and the differences for the traits of RRS and SSC were significant (*p* < 0.03). This indicates that chilling tolerance in the testcrosses is related to geographical distribution of the inbred lines. Correlations among the traits in the F_2:3_ population were similar to those in the association mapping panel. The two traits related to water status under chilling stress, LRD and WCS, were also significantly correlated with one another (*r* = 0.29). However, the correlation coefficients among the other traits were negative (−0.30 < *r* < −0.03).

### Genome-wide association mapping for the four chilling tolerance traits

Single nucleotide polymorphism (SNP) genotyping of the inbred lines (including Mo17) was conducted in a previous study^19^, and 556,809 high-quality SNPs with minor allele frequencies greater than 0.05 were present in the association mapping panel. In the entire population, a total of 19 significant SNP-trait associations were identified for the four traits with a threshold of *P* < 9.0 × 10^−6^, and individual significant SNP explained 6.53% to 9.06% of phenotypic variation (Table 2). These SNPs were distributed on chromosome 1, 2, 3, 4, 6, and 10. At 12 of the 19 significant loci heterozygous alleles had positive effects, and at six of the loci the trait means in genotypes with heterozygous alleles were similar to the trait means in genotypes with homozygous alleles (Table 2).

**Table 2:** Significant loci for the four chilling tolerance traits identified by GWAS across the testcrosses.

Trait	Chr^a^	Position^a^	Allele^b^	MAF^c^	P value	PVE (%)^d^	Mean^e^	
							MM	M_
LRD	1	227059212	C/A	0.50	3.75E-06	7.00	3.34	3.76^•^
	3	172905849	G/C	0.09	5.51E-06	6.59	3.48	4.12^•^
	4	236170393	G/A	0.32	5.26E-06	7.11	3.71	3.24
RRS	1	262720121	G/T	0.49	5.34E-06	6.90	0.96	1.08^•^
	2	226700684	C/T	0.21	8.01E-06	6.56	0.99	1.06^•^
	6	55648369	G/A	0.41	9.66E-07	7.55	0.98	1.08^•^
	6	55878334	G/A	0.35	8.14E-06	6.75	0.98	1.09^•^
	6	71234372	G/A	0.33	3.32E-06	6.75	0.98	1.11^•^
	10	142093709	T/A	0.25	1.27E-06	7.67	0.99	1.12^•^
SSC	2	5125992	C/T	0.05	2.57E-06	7.30	5.15	7.07^•^
	2	179981531	C/G	0.46	7.21E-06	6.98	4.91	5.75^•^
	3	210273085	A/T	0.14	5.37E-06	6.79	5.07	6.59^•^
WCS	1	207321533	G/T	0.09	4.82E-07	8.23	88.36	85.73^○^
	1	299902213	G/A	0.06	4.61E-06	7.00	85.15	88.32^•^
	2	225648692	C/A	0.06	3.32E-06	7.02	88.28	85.81^○^
	2	232102325	A/G	0.06	1.19E-06	7.57	88.28	85.71^○^
	3	3399413	A/G	0.08	1.31E-07	9.06	88.34	84.68^○^
	3	167810337	G/C	0.12	7.95E-06	6.63	88.4	86.24^○^
	6	158949497	C/T	0.06	8.80E-06	6.53	88.34	85.54^○^

^a^Loci revealed by GWAS. ^b^The minor alleles are underlined, and the Mo17 alleles are on the left of the slash sign; ^c^MAF = minor allele frequency; ^d^PVE = phenotypic variation explained by the locus; ^e^Means of the four traits in different genotypes at the significant loci; MM denotes the homozygous Mo17 alleles, and M_ is heterozygous. ^•^Heterozygous alleles at the significant loci had positive effects compaired to homozygous alleles; ^○^Heterozygous and homozygous alleles had similar effects.

The 338 inbred lines used for producing the testcrossing hybrids can be divided into the stiff stalk (SS), non-stiff stalk (NSS), and tropical-subtropical (TST) subpopulations according to a previous study^19^. Inbred lines in the first two subpopulations are temperate maize from two different heterotic groups. A total of 2, 10, and 1 significant loci for the four traits were revealed within the three subpopulations at the threshold of *P* < 1.810–6, respectively (Table 3). Individual locus explained 0.86–13.93% of phenotypic variation. In the SS subpopulation, the SNP (chr1: 53, 174, 087) was significantly associated with both LRD and RRS. Of the significant SNPs identified in the NSS subpopulation, the SNP (chr2: 1, 317, 451) was related to both SSC and WCS, and the locus (chr8: 82, 060, 785) simultaneously controlled LRD and RRS. At 10 of the 13 significant loci genotypes, heterozygous alleles had higher or similar trait means compared to the genotypes with homozygous alleles (Table 3).

**Table 3:** Subpopulation-specific significant loci for the four chilling tolerance traits.

Subpopulation	Trait	Chr^a^	Position^a^	Allele^b^	MAF^c^	P value	PVE (%)^d^	Mean^e^	
								MM	M_
SS	LRD	1	53174087	C/G	0.11	1.58E-06	13.93	2.17	3.71^•^
	RRS	1	53174087	C/G	0.11	1.44E-06	5.30	1.29	1.02
NSS	LRD	8	82060785	A/G	0.16	8.88E-08	1.55	3.52	3.93^•^
	RRS	2	231993006	A/G	0.05	2.49E-07	1.03	1.02	1.15^•^
		4	152476853	G/A	0.05	1.23E-06	5.22	1.05	0.83
		7	127719922	T/C	0.11	1.62E-06	0.86	1.04	0.98^○^
		8	82060785	A/G	0.11	8.48E-07	8.06	1.04	0.83
	SSC	2	1317451	A/C	0.08	1.03E-06	1.40	4.95	5.77^•^
	WCS	2	1317451	A/C	0.08	1.01E-06	0.90	88.29	87.02^○^
		3	171752201	A/G	0.09	9.73E-07	1.65	88.21	89.57^•^
		3	215466782	T/C	0.11	1.74E-06	5.47	88.11	90.80^•^
		6	106962973	C/T	0.05	1.15E-06	0.90	88.30	86.73^○^
TST	LRD	7	6218935	T/A	0.06	1.34E-06	3.14	3.30	4.04^•^

^a–e^See footnotes of the Table 2 for explanation.

### Candidate genes predicted according to the significant loci

The overall linkage disequilibrium (LD) decay distance was 50–100 kb when the *r* ^2^ cut-off value was set as 0.2^19^; thus, the candidate genes were predicted within the extension regions ranging from 50 kb upstream to 50 kb downstream of the significant SNPs. A total of 59 and 35 annotated genes were predicted according to the 19 and 13 loci identified by the entire population and subpopulations, respectively. Of these genes, 36 are putatively related to stress tolerance according to previous reports. These stress-related candidate genes can be classified into four categories according to their function. Detailed information for these genes is summarized in Supplemental Tables S1 and S2.

The first gene category includes four transcription factors and seven signal transduction genes. All of the candidate genes were predicted according to the loci identified from the entire population. *GRMZM2G110242* encodes a TEOSINTE BRANCHED/CYCLOIDEA/PCF (TCP) family transcription factor. Yang *et al*.^20^ found that down-regulating some TCPs enhanced cold tolerance in rice. Auxin responsive factors (ARFs) regulate the expression of auxin-responsive genes. *ARF3* (a *GRMZM2G437460* homolog) and *ARF4* were down-regulated by abiotic stresses and co-expressed with twenty-five abiotic stress-related genes^21^. Vascular plant One Zinc-finger proteins (VOZs) are plant-specific transcription activators. Loss-of-function mutations in *VOZ1* and *VOZ2* (a *GRMZM2G111696* homolog) increased cold and drought tolerance, and overexpression of *VOZ2* impaired freezing and drought tolerance in *Arabidopsis* ^22, 23^. *GRMZM2G110085* encodes a casein kinase 1 (CK1) protein. The CK1 family plays central roles in cellular stress-responses and carcinogenesis in animals^24^. *GMZM2G159756* encodes a member of wall-associated kinases (WAK), which have been suggested as extracellular environment sensors that triggers intracellular signals. The expression of *OsWAK98* (a *RMZM2G159756* homolog) changed under cold stress in rice^25^. *GRMZM2G058518* encodes a NAC gene, and NACs have been received much attention as regulators in various stress signaling pathways^26^. *GRMZM2G000936* encodes a BTB-related protein; its homolog in *Arabidopsis*, *EOL1*, acts collectively with *EOL2* to regulate ethylene biosynthesis by controlling ACC synthase levels^27^. *GRMZM2G403609* encodes a Rho GTPase activation protein (RhoGAP) with a pleckstrin homology. In rice, loss-of-function mutations in the RhoGAP protein SPIN6, which has a pleckstrin homology domain, led to programmed cell death and significantly elevated reactive oxygen species levels and defense-related gene expression^28^. *GRMZM2G411288* encodes a proline extensin-like receptor kinase (PERK), and PERK1 may be involved in the general perception and response to a wound and/or pathogenic stimulus in *B*. *napus* ^29^. *GRMZM2G053384* is a homolog of *AtLOI1*, which encodes a PPR protein involved in drought stress tolerance in *Arabidopsis* ^30^. *GRMZM2G470984* encodes a phytosulfokine (PSK). In *Arabidopsis*, expression of *AtPSK2* (homolog of *GRMZM2G470984*) was induced by fungal pathogens infection, and photosynthesis was significantly reduced in the knockout lines^31^.

The second category includes five transporter genes. *GRMZM2G363229* encodes a Multidrug and Toxic Compound Extrusion (MATE) efflux family protein. Zhang *et al*.^32^ found that a MATE member functioned as an ABA efflux transporter. Tonoplast intrinsic proteins (TIPs) are integral membrane proteins and function as aquaporins in plants. Zhu *et al*.^33^ found that *AtTIP2;3* (a *GRMZM2G027098* homolog) played an important role in drought tolerance in *Arabidopsis*. In plants, anion/ion channels and transporters are important for resistance to biotic and/or abiotic stresses^34, 35^. Of the candidate genes, *GRMZM2G463462* encodes a vacuolar iron transporter (VIT), *GRMZM2G332258* encodes a chloride channel protein and *GRMZM2G051917* is a plasma-membrane choline transporter gene.

Seventeen genes involving in metabolism processes related to stress tolerance were characterized as the third category. *GRMZM2G102927* and *GRMZM2G102811* encode class 1 glutamine-amidotransferases, a member of which, AtDJ-1a, confers stress protection through cytosolic SOD activation^36^. *GRMZM2G460383* encodes a carboxylesterase (CXE), and expression of *MdCXE1* and *MdCXE16* was upregulated by ethylene in apple^37^. *GRMZM2G035807* and *GRMZM2G092327* encode DEAD RNA helicase proteins, some of which played roles during stress adaptation processes in plants (reviewed by Jung *et al*.^38^). For example, RCF1, a cold-inducible RNA helicase, was found to be essential for maintaining proper pre-mRNA splicing and cold tolerance in *Arabidopsis* ^39^. *GRMZM2G457267* encodes a Sec20 family protein, and its homolog in *Arabidopsis* (*AtSec20*) is involved in osmotic stress tolerance^40^. ALA1 (a homolog of GRMZM2G407825 and GRMZM2G107841), a member of the aminophospholipid translocase family, is involved in chilling tolerance in *Arabidopsis* ^41^. Plant glutathione peroxidases (GPXs) protect cells from stress-induced oxidative damage (reviewed by Bela *et al*.^42^). *AtGPX1*, a *GRMZM2G012479* homolog, was upregulated in response to various abiotic stresses^43^, while reduced *AtGPX1* expression led to compromised photooxidative stress tolerance^44^. *GRMZM2G395535* encodes a glutaredoxin protein (GRX). GRXs act in antioxidant defense and some CC-type GRXs have roles in stress responses (reviewed by Gutsche *et al*.^45^). For example, class I Fe-S GRXs might constitute a sensor for oxidative stress conditions (reviewed by Couturier *et al*.^46^). *GRMZM2G053206* encodes an aspartyl protease. Overexpression of the ASPG1 (ASPARTIC PROTEASE IN GUARD CELL 1) gene can confer drought avoidance in *Arabidopsis* ^47^. UBP16 (a GRMZM2G000404 homolog) is an ubiquitin-specific protease, and its enzyme activity is required for salt tolerance^48^. *β*-carbonic anhydrases function in rapid CO_2_-induced stomatal movements; the *β*-carbonic anhydrase gene *PgCA* (a *GRMZM2G121878* homolog) was found to respond to various abiotic stresses in *Pennisetum glaucum* ^49^. Plant growth often reduces in response to environmental stresses. Three genes related to plant growth or development are in this category, including two genes related to DNA replication (*GRMZM2G032209*) and DNA repair (*GRMZM2G138161*) and one gene (*GRMZM2G082097*) associated with small RNA degradation nucleases.

Three genes involved in saccharometabolism are regarded as the fourth category. These genes encode a phosphoenolpyruvate carboxykinase (*GRMZM2G580389*), a phosphofructokinase (*GRMZM2G132882*) and a UTP-glucose-1-phosphate uridylyltransferase (*GRMZM2G019986*). They are key regulators in the soluble sugar metabolism pathway. Some soluble sugars were found to be significantly correlated to heterosis in freezing tolerance in *Arabidopsis* ^50^.

### Gene expression assay for some candidate genes under chilling stress

**Figure 1:**
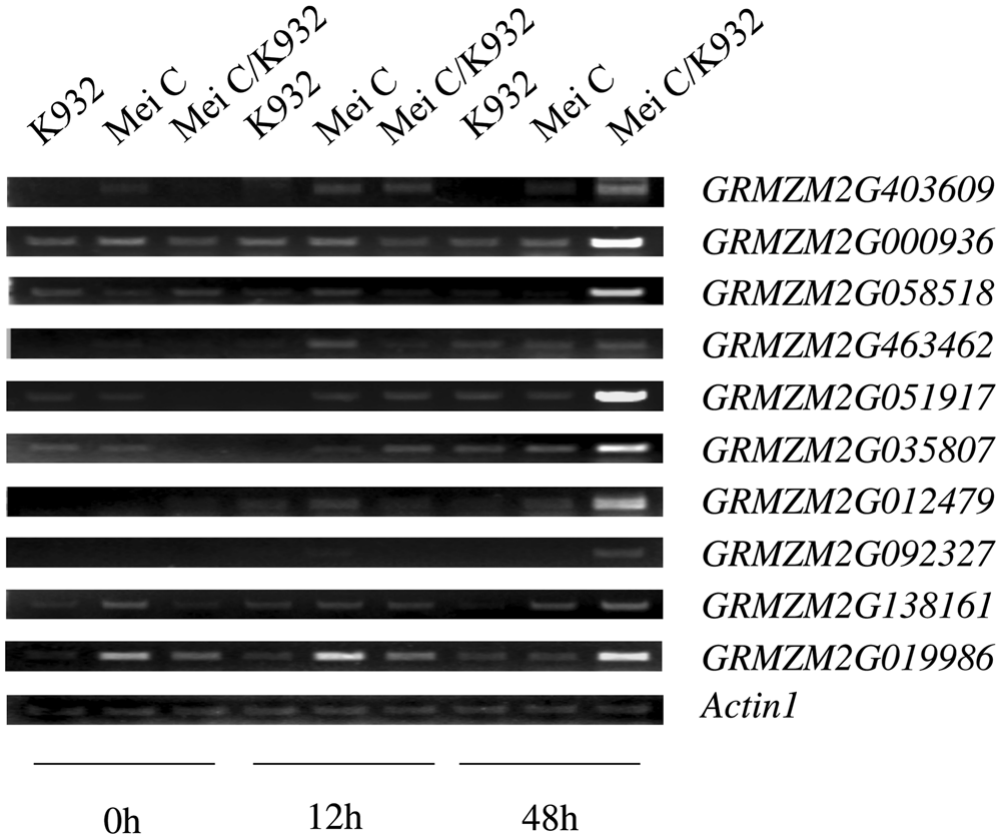
Expression of the 10 candidate genes in the two parents and their hybrid under chilling stress.

Two inbred lines, Mei C and K932, and their hybrid, Mei C/K932, were used to investigate the expression of the candidate genes under chilling stress in the growth chambers for 0, 12, and 48 hours (Fig. 1). Seedling culture and chilling stress treatment were the same as for the GWAS panel, and the experiment was laid out according to a randomized complete block design with two replicates. The experiment was repeated twice.

LRD was investigated one week after chilling stress. LRD were significantly higher in Mei C and the hybrid (Mei C/K932) than in the K932 parent, while the LRD between the Mei C and hybrid were not significant. This is consistent with the results observed in the field experiment conducted in the winter of 2014 (Fig. S1).

PCR primers were designed for the 36 candidate genes for the gene expression assay. Ten pairs of primers (Supplemental Table S3) successfully amplified the expected products in the two parents and their hybrid were then used to conduct the gene expression assay. In general, expression of the candidate genes was induced to different degrees by chilling stress.

Differential gene expression was observed between the parents and their hybrid under chilling stress. Expression of the ten candidate genes investigated in the hybrid apparently increased under chilling treatment for 48 h, except for *GRMZM2G463462* and *GRMZM2G138161* (Fig. 1). Moreover, *GRMZM2G035807* expression was also increased in the hybrid under chilling stress for 12 h. In the two parents, gene expression in the sensitive parent, K932, was not or slightly induced by chilling stress, while the expression of *GRMZM2G463462* and *GRMZM2G012479* was increased under chilling stress in the tolerance parent, Mei C (Fig. 1). Upregulation of these candidate genes in the hybrid or in only one of its parent under chilling stress indicates that these candidate genes could be involved in chilling tolerance heterosis in maize seedlings.

### The linkage map and QTLs for chilling tolerance revealed in the F_2:3_ population

The linkage map was constructed using a Mapmaker analysis based on data from 152 codominant, evenly distributed simple sequence repeat (SSR) markers assayed from 207 F_2:3_ families. The map covered a total length of 1958.2 cM, with an average interval of 12.9 cM between the adjacent markers.

QTLs for the four traits are listed in Table 4. A total of seven QTLs were resolved for the four traits; individual QTL explained 10.55–25.29% of phenotypic variation. For the additive effects, alleles from the tolerance parent, Mei C, had positive effects on the performance of these traits at five of the QTLs (Table 4). Interestingly, one region on chromosome 1, umc1774-phi26545-umc1862, was detected to have a significant effect on both RRS and SSC.

**Table 4:** QTLs for the four chilling tolerance traits resolved using composite interval mapping in the F_2:3_ population.

Trait	Chr^a^	Interval^a^	LOD	A^b^	D^**c**^	D/A^**d**^	PVE (%)^e^
LRD	1	umc1282-umc1948	5.47	0.17	0.60	OD	20.49
WCS	3	bnlg1957-umc1102	3.58	0.60	−1.20	OD	10.55
RRS	1	phi265454-umc1862	5.45	−0.09	−0.05	PD	14.10
	2	bnlg1316-umc1230	6.31	0.16	−0.09	PD	16.49
	3	umc1495-umc1717	4.20	−0.14	0.06	PD	10.84
SSC	1	umc1774-phi265454	7.00	1.42	0.71	PD	25.29
	1	umc1862-umc1630	7.71	1.74	−0.07	A	24.78

^a^Chromosome number and marker intervals; ^b^Additive effects - the positive values indicate the alleles from Mei C have the effect on increasing the trait value; ^c^Dominance effects - positive values indicate that heterozygotes have higher phenotypic values than the respective means of the two homozygotes; ^d^Gene action - OD = overdominance, PD = partial dominance, and A = additive; ^e^PVE = phenotypic variation explained by the locus.

Although the F_2:3_ family means usually underestimate dominance, partial dominance and overdominance were nonetheless still prevalent among the QTLs (Table 4). Two of the QTLs showed overdominance, four QTLs showed partial dominance, and only one QTL for SSC had additive effect.

## Discussion

Chilling at the maize seedling stage will weaken the seedlings and result in an eventual yield loss. A number of genetic studies have been conducted to identify genetic loci for chilling tolerance at the seedling stage by linkage mapping^9–13^ and association mapping with diverse inbred lines in maize^4, 5, 14^. Heterosis is one of the most important tools in plant breeding and has been demonstrated in plant stress tolerance^50^. Dissection of heterotic loci for yield and yield components has been extensively conducted^18, 51, 52^, though seldom has been addressed on stress tolerance. Thus, it is urgent to determine the genetic basis and dissect the genetic loci associated with heterosis for chilling tolerance in crops like maize and rice, where hybrids are extensively used.

Instead of inbred lines, a GWAS with hybrids or testcrosses might identify genetic loci related to heterosis. Huang *et al*.^18^ have demonstrated that a GWAS with rice hybrids could detect superior alleles that contribute to heterosis. In this study, we conducted a GWAS for chilling tolerance at the seedling stage with testcrossing hybrids in maize. In total, 32 significant loci were identified, and 36 candidate genes that are potentially associated with stress tolerance were predicted based on these loci. Among these candidate genes, two *GRMZM2G111696* and *GRMZM2G407825*/*GRMZM2G107481* homologs in *Arabidopsis*, *ATVOZ2* and *ALA1*, have been confirmed to be involved in chilling or freezing tolerance^22, 23, 41^. Additionally, homologs for 19 candidate genes in *Arabidopsis* or rice (Supplemental Tables S1 and S2) have been reported to be associated with tolerance to other abiotic stresses. For example, in *Arabidopsis*, *AtLOI1* (a *GRMZM2G053384* homolog), *AtSec20* (a *GRMZM2G457267* homolog), and *AtTIP2;3* (a *GRMZM2G027098* homolog) were found to be involved in osmotic or drought tolerance^30, 33, 40^, while *AtCLC-C* (a *GRMZM2G332258* homolog) and *UBP16* (a *GRMZM2G000404* homolog) were associated with salt tolerance^34, 48^. However, no locus or candidate gene is identical to that revealed by GWAS with maize inbred lines in previous studies^4, 5, 14^. Near the significant loci (chr3: 210273085) harboring the candidate gene *GRMZM2G058518*, Presterl *et al*.^13^ also identified a QTL for fresh matter yield under chilling stress by linkage mapping with testcrosses. It is noteworthy that genotypes with heterozygous alleles at most of the significant loci had higher or similar trait values compared to homozygous alleles. Additionally, in comparison with their parents, chilling stress apparently induced the expression of the candidate genes in the hybrid. In previous studies, gene expression profile comparisons between the hybrids and their parents also revealed a number of heterosis-related genes^53, 54^, including a maize gene that increased leaf size in *Arabidopsis* ^54^. Thus, the genetic loci and candidate genes revealed in this study are more likely related to heterosis. These significant loci and candidate genes should be important for genetic improvement regarding chilling tolerance in maize hybrids.

F_2_ populations or F_2:3_ families have been employed to identify genetic factors that contribute to heterosis; dominance, overdominance and epistasis were found to be important contributors to heterosis in yield and yield-related traits^51, 52, 55^. In this study, QTL mapping with an F_2:3_ population derived from the two parents used for the gene expression assay revealed seven QTLs for chilling tolerance. Six of them showed partial dominance and overdominance, suggesting that partial dominance and overdominace play an important role in heterosis for chilling tolerance at the seedling stage. Fracheboud *et al*.^10^ identified 19 QTLs for nine photosynthesis-related traits under chilling stress (15 °C) and 14 stable QTLs across different environments with an F_2:3_ population. Most of the QTLs (29) displayed overdominance, partial dominance or dominance effects. In the phi26546-umc1862 region on chromosome 1, which harbored a QTL for RRS in this study, Fracheboud *et al*.^10^ also detected a QTL with an overdominance effect. This further indicates that heterosis plays an important role in chilling tolerance in maize seedlings and that the QTLs related to heterosis could be important for maize hybrid breeding.

Lv *et al*.^15^ found the cold adaptability in rice is associated with subpopulation and latitudinal distribution. In this study, chilling tolerance in the testcrosses derived from the temperate subpopulation was better than that in the testcrosses from the tropical and subtropical subpopulations. Moreover, the significant SNPs revealed by the GWAS across all testcrossing hybrids were different from those identified by the GWAS within the subpopulations in this study. Strikingly, half (11/22) of the candidate genes predicted based on the significant SNPs identified by the entire population are mainly transcriptional factors and signal transduction genes, while most of the candidate genes predicted according to the subpopulation-specific SNPs are involved in metabolism pathways related to stress tolerance. This indicates that the GWAS within subpopulations could identify additional significant loci and different types of candidate genes.

## Materials and Methods

### Plant materials

The testcrossing association mapping population was produced by crossing a CMS-S line (S-Mo17) with 338 diverse inbred lines (55, 119 and 164 belong to the SS, NSS and TST subpopulations, respectively). The inbred lines have been genotyped with 556,809 SNPs^19^. The CMS-S line, S-Mo17, was developed by the maize research group at Huazhong Agricultural University and has the same nuclear background as inbred line Mo17.

The F_2:3_ population consisting of 207 families was developed from a cross between K932 and Mei C. K932 is a chilling-sensitive maize inbred line that was provided by the Heilongjiang Academy of Land Reclamation of Sciences, and Mei C is a chilling tolerance inbred line developed by the Shiyan Academy of Agriculture in Hubei province, China.

### Seedling culturing and chilling treatment

Seedling culturing and chilling treatments were similar to the experiments described by Huang *et al*.^14^. The experiments were conducted in growth chambers (HP400GS, Ruihua, Wuhan, China) for the testcrossing association mapping panel and the F_2:3_ population. The population and parent seeds were directly sown into patented seedling hydroponic cultivation boxes (ZL200920177285.0, China). For the GWAS experiment, a completely randomized design with three replicates was employed, and each replicate contained 5 seedlings with a space of 19.5 cm between the adjacent rows. Chilling tolerance experiment for the F_2:3_ population was also conducted in the growth chambers with the same method, except that only two replicates (10 seedlings in each replicate) were adopted. The seeds were socked in tap water for 24 hours, germinated and then grown in the hydroponic cultivation boxes at 25–26 °C. As the coleoptiles were emerging, Hoagland solution was added and replaced every 3 days. Chilling stress (5.5–6.5 °C) was applied for 7 days at approximately the 3-leaf stage.

### Traits measurements

The four chilling tolerance-related traits, LRD, WCS, RRS, and SSC, were investigated in the association mapping and F_2:3_ populations. The traits were investigated immediately after 7 days of chilling stress treatment. LRD was scored with a range from 1 (totally rolled) to 5 (not rolled at all). Roots and shoots (upground) from the plants in each replicate were separated weighed, and then immediately oven-dried at 105 °C for 1 hour and maintained at 80 °C for 24 hours. The dry weights of the shoots (upground) and roots were determined. WCS (%) was calculated as the percentage of (fresh upground weight - dry upground weight)/fresh upground weight, and RRS was computed as the ratio of fresh upground weigh/fresh root weight. Oven-dried shoots and leaves in each replicate were ground and the SSC (%) was measured with the anthrone colorimetric method described by Li *et al*.^56^.

### RNA extraction and RT-PCR analysis

RNA from the leaves of the seedlings treated with chilling stress (5.5–6.5 °C) in the growth chambers for 0 h, 12 h, and 48 h was isolated with the Trizol reagent (Invitrogen, Carlsbad, CA, USA). Total RNA was reverse transcribed using the TransScript OneStep gDNA Removal and cDNA Synthesis SuperMix Kit (TransGen Biotech, Beijing, China) according to the manufacturer’s instructions. The semi-quantitative RT-PCR was performed by initially denaturing template cDNA at 95 °C for 5 min, then 26–33 cycles at 95 °C for 40 sec, 53–55 °C for 40 sec, and 72 °C for 40 sec; and a final extension at 72 °C for 5 min. Each experiment had two biological replicates. The Actin1 gene *GRMZM2G126010* was used as the reference gene.

### Statistical analysis and association mapping

ANOVA and correlation analyses were performed with generalized linear modeling (GLM) and correlation (CORR) procedures using the SAS program (Release 9.1.3; SAS Institute, Cary, NC, USA). SNP genotyping of the inbred lines, population structure (Q matrix) and relative kinship matrix (K) were conducted in a previous study^19^. In total, 556,809 high quality SNPs with minor allele frequencies greater than 0.05 were employed for the GWAS. The GWAS was conducted with the TASSEL V4.0^57^ software package across the testcrosses (with the GLM + Q model) and within each subpopulation (with the MLM + Q + K model). For the GWAS across the testcrosses, a Bonferroni-corrected threshold at α = 5 (n = 556,809, *P*_5/n_ < 9.0 × 10^−6^) was used to declare a significant SNP-trait association for all the traits. For subpopulation-specific SNP-trait associations, a threshold at α = 1 (n = 556,809, *P*_1/n_ < 1.8 × 10^−6^) was employed, as fewer testcrosses were included in each subpopulation. The positions of the SNPs at the identified loci for the corresponding traits were based on the public maize genome data set B73 RefGen_v2. The mean effects of either homozygous genotypes (MM, homozygous Mo17 allele) or heterozygous genotypes (M_) at each significant locus were calculated according to the genotype of Mo17.

### DNA markers, map construction and QTL analysis

A total of 152 codominant, evenly distributed nuclear SSR markers were selected to construct the linkage map. The program of Mapmaker/EXP 3.0^58^ was used to construct the genetic linkage map. The means of the traits were used to identify the QTLs with Windows QTL Cartographer 2.5^59^. The LOD thresholds for the traits were determined from 500 random permutations.

## Electronic supplementary material

Supplementary Information
