# Supplementary material for: Genetic mapping with testcrossing associations and F2:3 populations reveals the importance of heterosis in chilling tolerance at maize seedling stage
Source: Sci Rep. 2017 Jun 12;7:3232. doi: 10.1038/s41598-017-03585-0 (PMC5468334; doi:10.1038/s41598-017-03585-0)
Supplement: Supplementary file 1 — Supplementary Information [file 41598_2017_3585_MOESM1_ESM.pdf]

Genetic mapping with testcrossing association and $F_{2:3}$ populations reveals the importance of heterosis in chilling tolerance at maize seedling stage

Jinbo Yan[1#], Yu Wu[1#], Wenming Li[1], Xiner Qin[1], Yi Wang[2], Bing Yue[1,*]

1 National Key Laboratory of Crop Genetic Improvement, Huazhong Agricultural University, Wuhan, China

2 Industrial Crops Research Institution, Heilongjiang Academy of Land Reclamation of Sciences, Haerbin, China

[#] These authors contributed equally to this work.

[*] **Corresponding author:** Bing Yue

Email: yuebing@mail.hzau.edu.cn,

Phone: +86-27-87286870

Supplementary figure, table and data file legends

Supplementary Figure

Figure S1 Performance of chilling tolerance in Mei C, K932 and their hybrid

A Leaf rolling degree in the two parents and their hybrid in the field experiment of 2014 winter

B Leaf rolling degree in the two parents and their hybrid in the experiment of growth chamber

Supplementary Tables

Supplemental table 1 Annotation and category of the candidate genes predicted according to the significant loci revealed by GWAS across all the testcrosses

Supplemental table 2 Annotation and category of the candidate genes predicted according to the subpopulation-specific significant loci

Supplemental table 3 Sequences of the ten pairs of primers used for gene expression assay in this study

Supplementary Figure

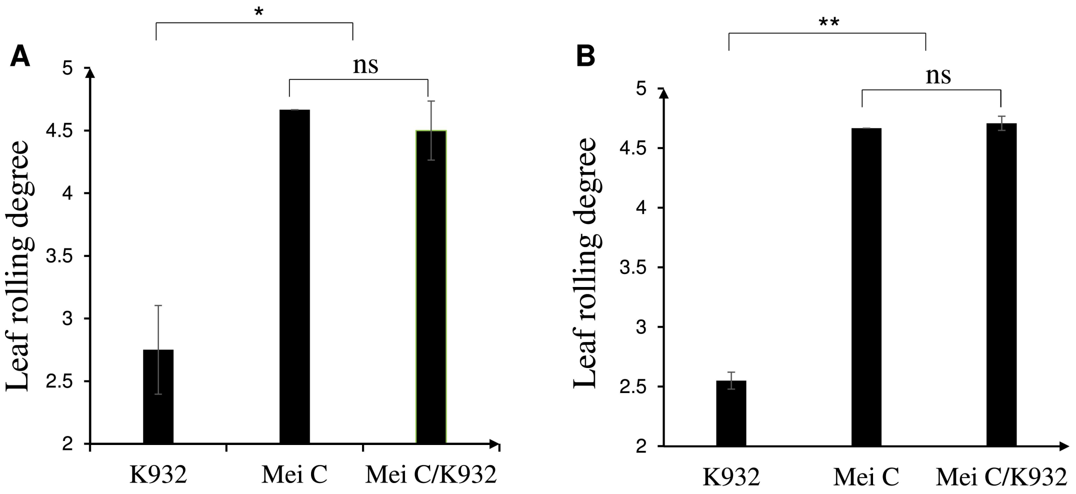

Supplementary Figure S1

Supplementary Tables

Supplemental table 1 Annotation and category of the candidate genes predicted according to the significant loci revealed by GWAS across all the testcrosses

Chr	Position [a]	Predicted genes	Putative function [b]	Category
1	207321533	GRMZM2G460383	alpha/beta-Hydrolases superfamily protein；CXE carboxylesterase	3
	299902213	GRMZM2G363229	MATE efflux family protein；MATE efflux family protein putative expressed	2
2	5125992	GRMZM2G082097	(SDN3) small RNA degrading nuclease 3	3
		GRMZM2G032209	(ATCDT1A, CDT1, CDT1A) homolog of yeast CDT1 A；CDT1B - Putative DNA replication initiation protein	3
	179981531	GRMZM2G110242	(ATTCP18, BRC1, TCP18) TCP family transcription factor	1
	225648692	GRMZM2G159756	protein serine/threonine kinases;OsWAK98-OsWAK receptor-like cytoplasmic kinase	1
		GRMZM2G470984	(ATPSK2, PSK2) phytosulfokine 2 precursor	1
	226700684	GRMZM2G053384	(LOI1, MEF11) Pentatricopeptide repeat (PPR) superfamily protein	1
		GRMZM2G000936	(EOL1) ETO1-like 1；BTBT3-Bric-a-Brac Tramtrack Broad Complex BTB domain with tetratricopeptide repeats	1
	232102325	GRMZM2G035807	P-loop containing nucleoside triphosphate hydrolase; DEAD-box RNA helicase	3
3	3399413	GRMZM2G102927	Class I glutamine amidotransferase-like superfamily protein；DJ-1 family protein	3
		GRMZM2G102811	Class I glutamine amidotransferase-like superfamily protein；DJ-1 family protein	3
	167810337	GRMZM2G457267	(AtSec20) Sec20 family protein；MPPN domain containing protein	3
	172905849	GRMZM2G332258	(ATCLC-C, CLC-C) chloride channel C；chloride channel protein	2
		GRMZM2G110085	Protein kinase family protein；CK1_CaseinKinase_1a.6 - CK1 includes the casein kinase 1 kinases expressed	1
	210273085	GRMZM2G058518	(ANAC073, NAC073, SND2) NAC domain containing protein 73	1
		GRMZM2G437460	(ARF3, ETT) Transcriptional factor B3 family protein/auxin-responsive factor AUX/IAA-related	1
4	236170393	GRMZM2G580389	phosphoenolpyruvate carboxykinase	4
6	55648369	GRMZM2G463462	Vacuolar iron transporter (VIT) family protein	2
	55878334	GRMZM2G403609	(REN1) Rho GTPase activation protein (RhoGAP) with PH domain；pleckstrin homology domain-containing protein	1
		GRMZM2G132882	Phosphofructokinase family protein	4
	158949497	GRMZM2G111696	(ATVOZ2, VOZ2) vascular plant one zinc finger protein 2	1
		GRMZM2G411288	(ATPERK1, PERK1) proline extensin-like receptor kinase 1	1
10	142093709	GRMZM2G019986	UTP--glucose-1-phosphate uridylyltransferase	4

[a] The positions of the candidate genes were based on the public maize genome data set B73 RefGen_v2.

[b] The homologs of the candidate genes in red have been reported associating with stress tolerance in *Arabidopsis* or rice.

Supplemental table 2 Annotation and category of the candidate genes predicted according to the subpopulation-specific significant loci

Chr	Position [a]	Predicted genes	Putative function [b]	Category
**SS subpopulation**				
1	53174087	GRMZM2G407825	(ALA1) aminophospholipid ATPase 1; phospholipid-transporting ATPase	3
		GRMZM2G107481	(ALA1) aminophospholipid ATPase 1; phospholipid-transporting ATPase	3
**NSS subpoplation**				
2	1317451	GRMZM2G051917	Plasma-membrane choline transporter family protein	2
	231993006	GRMZM2G053206	Eukaryotic aspartyl protease family protein；xylanase inhibitor putative expressed	3
		GRMZM2G092327	P-loop containing nucleoside triphosphate hydrolases superfamily protein; DEAD-box RNA helicase	3
3	171752201	GRMZM2G138161	DNA repair metallo-beta-lactamase family protein	3
	215466782	GRMZM2G348512	(BETA CA2, CA18, CA2) carbonic anhydrase 2	4
		GRMZM2G121878	(BETA CA2, CA18, CA2) carbonic anhydrase 2	4
4	152476853	GRMZM2G027098	(ATTIP2;3, DELTA-TIP3, TIP2;3) tonoplast intrinsic protein 2;3；aquaporin protein	2
6	106962973	GRMZM2G012479	(ATGPX1, GPX1) glutathione peroxidase 1	3
7	127719922	GRMZM2G000404	(UBP16) ubiquitin-specific protease 16；ubiquitin carboxyl-terminal hydrolase domain containing protein	3
**TST subpopulation**				
7	6218935	GRMZM2G395535	Glutaredoxin family protein；glutaredoxin putative expressed	3

[a] The positions of the candidate genes were based on the public maize genome data set B73 RefGen_v2.

[b] The homologs of the candidate genes in red have been reported associating with stress tolerance in *Arabidopsis* or rice.

Supplemental table 3 Sequences of the primers used for gene expression assay

Gene name	Forward primer (5′-3′)	Reverse primer (5′-3′)
GRMZM2G403609	ACACCCATGATGACGATGGT	CTCTTCAGCTGGTCCCTCAA
GRMZM2G000936	GCGAGGAAGGATTGCAGAAG	ACCAGCTCTGTTGTTCCGTA
GRMZM2G058518	CACCGGTGGAAAACTCAAGG	GCACCTCAAAGTTAGGCACC
GRMZM2G463462	GCTGAGAGGGATGAATGGGA	GGAGCTGCTGAATCCAATGG
GRMZM2G051917	AGCCTTGGATCTGTTGCTCT	TCTTGTTGTGTGCGGACTTG
GRMZM2G035807	GGAGACAGGACACAACAGGA	TGATCCGCCATATCCTCCAC
GRMZM2G012479	ATGGCTCCCTCGTTCACTAG	TTTGCTGTTGTCAACCCACA
GRMZM2G092327	CAACTCCTGGTCGCTTGATG	TCCTGTTGTGTCCTGTCTCC
GRMZM2G138161	ATTTGTGCCACCTCAACCAC	TGACAAGCGATGGAAGAGGT
GRMZM2G019986	TCCCCATCCACAGAGCTTTT	ATCCGAACCTGACCCATTGT
Actin1/GRMZM2G126010	TACGAGATGCCTGATGGTCAGGTCA	TGGAGTTGTACGTGGCCTCATGGAC
